# Relationship between native papillary muscle T_1_ time and severity of functional mitral regurgitation in patients with non-ischemic dilated cardiomyopathy

**DOI:** 10.1186/s12968-016-0301-y

**Published:** 2016-11-16

**Authors:** Shingo Kato, Shiro Nakamori, Sébastien Roujol, Francesca N. Delling, Shadi Akhtari, Jihye Jang, Tamer Basha, Sophie Berg, Kraig V. Kissinger, Beth Goddu, Warren J. Manning, Reza Nezafat

**Affiliations:** 1 Department of Medicine (Cardiovascular Division), Beth Israel Deaconess Medical Center and Harvard Medical School, 330 Brookline Avenue, Boston, MA 02215 USA; 2 Department of Radiology, Beth Israel Deaconess Medical Center and Harvard Medical School, Boston, MA USA; 3 Department of Cardiology, Yokohama City University Hospital, Yokohama, Japan; 4 Biomedical Engineering Department, King’s College London, London, UK; 5 Biomedical Engineering Department, Cairo University, Giza, Egypt

## Abstract

**Background:**

Functional mitral regurgitation is one of the severe complications of non-ischemic dilated cardiomyopathy (DCM). Non-contrast native T_1_ mapping has emerged as a non-invasive method to evaluate myocardial fibrosis. We sought to evaluate the potential relationship between papillary muscle T_1_ time and mitral regurgitation in DCM patients.

**Methods:**

Forty DCM patients (55 ± 13 years) and 20 healthy adult control subjects (54 ± 13 years) were studied. Native T_1_ mapping was performed using a slice interleaved T_1_ mapping sequence (STONE) which enables acquisition of 5 slices in the short-axis plane within a 90 s free-breathing scan. We measured papillary muscle diameter, length and shortening. DCM patients were allocated into 2 groups based on the presence or absence of functional mitral regurgitation.

**Results:**

Papillary muscle T_1_ time was significantly elevated in DCM patients with mitral regurgitation (*n* = 22) in comparison to those without mitral regurgitation (*n* = 18) (anterior papillary muscle: 1127 ± 36 msec vs 1063 ± 16 msec, *p* < 0.05; posterior papillary muscle: 1124 ± 30 msec vs 1062 ± 19 msec, *p* < 0.05), but LV T_1_ time was similar (1129 ± 38 msec vs 1134 ± 58 msec, *p* = 0.93). Multivariate linear regression analysis showed that papillary muscle native T_1_ time (β = 0.10, 95 % CI: 0.05–0.17, *p* < 0.05) is significantly correlated with mitral regurgitant fraction. Elevated papillary muscle T_1_ time was associated with larger diameter, longer length and decreased papillary muscle shortening (all *p* values <0.05).

**Conclusions:**

In DCM, papillary muscle native T_1_ time is significantly elevated and related to mitral regurgitant fraction.

## Background

Functional mitral regurgitation, a consequence of left ventricular (LV) remodeling despite normal mitral valve structure, is one of the common and severe complications in non-ischemic dilated cardiomyopathy patients (DCM) [1–4]. It has been reported that the annular enlargement and mitral leaflet tethering by the displacement of papillary muscles due to LV dilatation are the main mechanisms of functional mitral regurgitation [5]. To date, little is known regarding the relationship between papillary muscle function and mitral regurgitant fraction in DCM patients.

Native (non-contrast) T_1_ mapping has emerged as a cardiovascular magnetic resonance (CMR) method to assess LV diffuse myocardial fibrosis [6]. Studies have shown that native T_1_ mapping detects diffuse myocardial abnormalities in hypertrophic cardiomyopathy and DCM [7–9]. The extent of myocardial damage by acute myocardial infarction can also be accurately assessed by native T_1_ mapping [10]. Diffuse myocardial abnormalities in patients with cardiac amyloidosis [11] and Anderson-Fabry disease [12, 13] can be assessed by native T_1_ mapping. However, data is lacking regarding native T_1_ mapping of papillary muscles in these myopathies, including DCM.

Therefore, we sought to examine papillary muscle native T_1_ mapping in DCM and to investigate the relationship between papillary muscle native T_1_ time and functional mitral regurgitation in this population.

## Methods

### Study subjects

Forty DCM patients (55 ± 13 years; 31 male) and 20 healthy adult control subjects (54 ± 13 years; 13 male) free of any cardiovascular diseases were studied. All participants were in sinus rhythm at the time of scan.

### CMR acquisition

CMR was performed using a 1.5 T system and a 32-channel cardiac phased array receiver coil (Achieva, Philips Healthcare, Best, The Netherlands). Cine CMR, phase contrast images of the ascending aorta, T_1_ mapping and 3 dimensional late gadolinium enhancement (LGE) were obtained in all participants [14]. T_1_ mapping was performed using a free-breathing slice-interleaved T_1_ (STONE) sequence [15].

Electrocardiogram monitoring leads were positioned with the subject in the supine position. Vertical and horizontal LV long-axis cine images were acquired using a steady-state free precession (SSFP) sequence. LV volumes and mass were calculated from an LV short-axis stack of cine images extending from the apex to the base (repetition time (TR), 3.3 ms; echo time (TE), 1.6 ms; flip angle (FA), 60°; field-of-view (FOV), 320 × 320 mm; acquisition matrix, 128 × 128; slice thickness, 8 mm; gap, 2 mm) [16]. Phase contrast images were acquired perpendicular to the proximal ascending aorta to quantify blood flow by following parameters (TR, 15 ms; TE, 6.5 ms; FA, 30°; velocity encoding, 300 cm/s; FOV, 320 × 320 mm; acquisition matrix, 288 × 288; number of phases per cardiac cycle, 36). STONE native T_1_ mapping was acquired in the short axis during free-breaching using slice-tracking with a balanced SSFP readout (5 slices, TR/TE = 2.8/1.4 msec, flip angle = 70, FOV = 360 × 351 mm, voxel size = 2.1 × 2.1 mm, slice thickness = 8 mm, TFE factor = 86, SENSE factor = 2).

Fifteen minutes after the injection of 0.2 mmol/kg gadobenate dimeglumine, LGE images were acquired using a 3 dimensional sequence [14] with following parameters (TR, 5.3 ms; TE, 2.1 ms; FA, 70°; FOV, 320 × 320 × 125 mm^3^; acquisition matrix, 224 × 224 × 23; spatial resolution,1.4 × 1.4 × 1.5 mm; reconstruction resolution, 0.6 × 0.6 × 0.8 mm).

### Image analysis

**Fig. 1:**
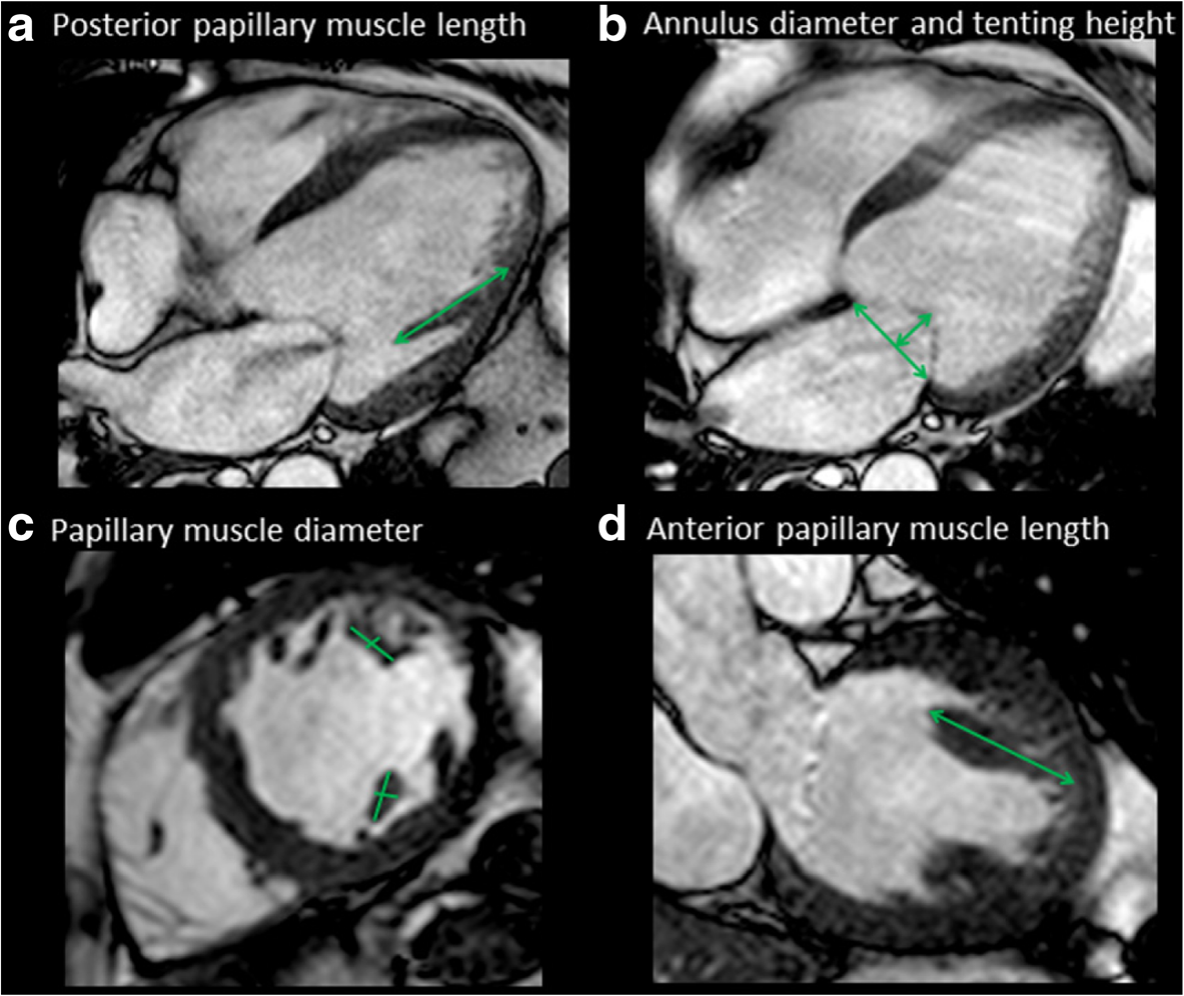
Measurement of papillary muscle, mitral annulus and tenting height. (**a**) posterior papillary muscle length measurement, (**b**) mitral annulus and tenting height measurement, (**c**) papillary muscle diameter measurement, (**d**) anterior papillary muscle length measurement

Data were analyzed using a commercial workstation (Extend MR WorkSpace, version 2.3.6.3, Philips Healthcare). To determine LV mass, epi- and endocardial LV borders were manually traced on the short axis images. LV mass was calculated as the sum of the myocardial volume multiplied by the specific gravity (1.05 g/mL) of myocardial tissue [17]. Left atrial (LA) volume was calculated using biplane area length method [18]. Papillary muscle morphology (diameter, length and shortening), anterior and posterior mitral leaflet length, mitral annulus diameter (both in 2 chamber and 4 chamber views) and tenting height were measured by cine CMR (Fig. 1). Papillary muscle shortening was calculated as follows.

$$ Papillary\  muscle\  shortening\ \left(\%\right)=\left[ maximum\  papillary\  muscle\  length\ (mm)\hbox{--} minimum\  papillary\  muscle\  length\ (mm)\right]/ maximum\  papillary\  muscle\  length\ (mm)\times 100 $$

Aortic blood flow was determined using the semi-automated algorithms [19]. Phase offset correction was performed as described previously [20]. Mitral regurgitation volume was calculated as the difference between the LV stroke volume and ascending aorta forward flow [21]. Mitral regurgitant fraction was calculated as follows.

$$ Mitral\  regurgitant\  fraction\ \left(\%\right)=\left[LV\  stroke\  volume\ (mL)\hbox{--} ascending\  aorta\  forward\  flow\ (mL)\right]/LV\  stroke\  volume\ (mL)\times 100 $$

**Fig. 2:**
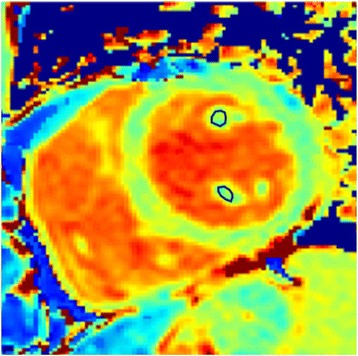
Measurement of native T_1_ time of papillary muscles. Location of anterior and posterior papillary muscle T_1_ measurement. *Blue circle* represents region of interest for T_1_ measurement. *ROI* region of interest

For calculating papillary muscle native T_1_ time, both anterior and posterior papillary muscles were manually traced on custom software (MediaCare, Boston, MA, USA) (Fig. 2). For calculating LV native T_1_ time, the three short axis LV slices were then divided into 6 segments for base and mid slices, 4 segments for apical slice using the anterior right ventricular-LV insertion point as reference. The 16 segment model was used to assess native T_1_ time in each segments. The native T_1_ time from all the segments was averaged to calculate each subjects LV T_1_ time. Motion correction was performed using the adaptive registration of varying contrast-weighted images for improved tissue characterization (ARCTIC) approach [22]. To evaluate inter-observer variability, measurements of papillary muscle native T_1_ time from 10 DCM patients and 10 healthy adult controls were independently taken by two observers. One of the two observers measured papillary muscle native T_1_ time twice to assess intra-observer variability. The time delay between two read for intra-observer variability was 1 month. Visual assessment was performed to evaluate for LV and papillary muscle scar on LGE.

### Statistical analysis

Data were analyzed using SPSS software (version 17.0, SPSS, Inc., Chicago, IL, USA) and MedCalc for Windows (version 14.8.1, MedCalc Software, Ostend, Belgium). Continuous values are presented as mean ± standard deviation (SD). Categorical values are expressed as number (%). DCM patients were divided into 2 groups based on the presence or absence of mitral regurgitation. To assess the difference between 3 groups (DCM patients with mitral regurgitation, *n* = 22; DCM patients without mitral regurgitation, *n* = 18; control subjects, *n* = 20), one-way analysis of variance (ANOVA) with Tukey’s correction was used for continuous variables. Chi-square test was used to assess the difference for categorical variables. Bland and Altman plot [23], coefficient of variation (CV) were used to evaluate intra- and inter-observer variability for measuring papillary muscle native T_1_ time. Repeatability coefficients were calculated as 1.96 times the SD of the differences on the Bland-Altman plots. Spearman’s correlation coefficient was calculated to evaluate relationship between papillary muscle T_1_ time and papillary muscle morphology (diameter, length and shortening). Multivariate linear regression analysis was performed to identify the determinants of mitral regurgitation severity in DCM patients. Variables with *p*-value <0.05 in the univariable analysis were included in the multivariable linear regression analysis (stepwise method). *P* value <0.05 was considered as statistically significant.

## Results

### Patients’ characteristics

Table 1 summarizes the clinical characteristics of study subjects. There was no significant difference in gender, age, body mass index, systolic and diastolic blood pressure, heart rate between 3 groups. Information of medical therapy was also shown in Table 1.

**Table 1:** Characteristics of study subjects

	DCM MR (+), *N* = 22	DCM MR (-), *N* = 18	Controls, *N* = 20	* P-value DCM MR (+) vs DCM MR (-)	* P-value DCM MR (+) vs Controls	* P-value DCM MR (-) vs Controls
Demographics						
Male, %	15 (68 %)	16 (89 %)	13 (65 %)	0.12	0.83	0.083
Age, years	52 ± 16	58 ± 13	54 ± 13	0.32	0.98	0.44
Height, cm	176 ± 9	175 ± 8	172 ± 8	0.99	0.41	0.52
Body weight, kg	93 ± 20	86 ± 15	79 ± 15	0.47	0.10	0.67
BMI, kg	30 ± 7	28 ± 6	27 ± 4	0.51	0.21	0.86
BSA, m^2^	2.1 ± 0.3	2.0 ± 0.2	1.9 ± 0.2	0.58	0.10	0.57
SBP, mmHg	116 ± 14	119 ± 17	119 ± 12	0.80	0.70	0.99
DBP, mmHg	70 ± 11	74 ± 14	71 ± 10	0.53	0.70	0.59
Heart rate, bpm	76 ± 12	73 ± 12	68 ± 14	0.77	0.14	0.47
Medications						
Aspirin	7 (32 %)	7 (39 %)	-	0.64	-	-
ACE/ARBs	18 (82 %)	16 (89 %)	-	0.38	-	-
Calcium channel blockers	2 (9 %)	2 (11 %)	-	0.83	-	-
Beta blockers	17 (77 %)	14 (78 %)	-	0.97	-	-
Diuretics	11 (50 %)	7 (39 %)	-	0.48	-	-
Aldosterone inhibitors	2 (9 %)	1 (6 %)	-	0.67	-	-
Statin	9 (41 %)	9 (50 %)	-	0.57	-	-
Warfarin	5 (8 %)	2 (11 %)	-	0.90	-	-

*P value was calculated by one-way ANOVA with Tukey’s correction or Chi-square test

*ACE* angiotensin converting enzyme inhibitor, *ANOVA* analysis of variance, *ARB* angiotensin receptor blocker, *BMI* body mass index, *BSA* body surface area, *DBP* diastolic blood pressure, *DCM* dilated cardiomyopathy, *HR* heart rate, *MR* mitral regurgitation, *SBP* systolic blood pressure

### Cine MRI and LGE findings

Table 2 summarizes CMR findings. LV end-diastolic volume index, LV end-systolic volume index, LV mass index were significantly higher (all *p* < 0.05) in DCM patients in comparison to controls. Stroke volume index and LV ejection fraction were significantly decreased (*p* < 0.05) in DCM patients compared to control subjects. LA volume was significantly higher in DCM patients with MR compared to those without MR (*p* = 0.04). LGE of LV myocardium was observed in 8 of 40 (20 %) DCM patients, while no DCM patients had LGE of the papillary muscles. Healthy control subjects did not show any LGE in the LV or the papillary muscles. Table 3 shows comparison of papillary muscle parameters. Papillary muscle was significantly thicker, longer in DCM patients in comparison to healthy controls. Regarding mitral annulus diameter, papillary muscle shortening and tenting height, significant difference was also observed between DCM patients with mitral regurgitation and those without mitral regurgitation. Table 4 summarizes the intra- and inter-observer variability for measurement of papillary muscle size. Intra class correlation coefficients for papillary muscle size measurement were >0.80 both for intra- and inter-observer variability.

**Table 2:** Comparison of CMR parameters

	DCM MR (+), *N* = 22	DCM MR (-), *N* = 18	Controls, *N* = 20	* P-value DCM MR (+) vs DCM MR (-)	* P-value DCM MR (+) vs Controls	* P-value DCM MR (-) vs Controls
Cine MRI parameters						
EDVI, mL/m^2^	129 ± 43	122 ± 36	80 ± 16	0.79	<0.05	<0.05
ESVI. mL/m^2^	89 ± 43	84 ± 36	32 ± 10	0.86	<0.05	<0.05
SVI, mL/m^2^	40 ± 11	38 ± 10	48 ± 10	0.86	0.04	0.02
LVEF, %	34 ± 13	33 ± 11	61 ± 4	0.98	<0.05	<0.05
LVMI, g/m^2^	67 ± 23	68 ± 17	46 ± 9	0.99	<0.05	<0.05
RVEF, %	52 ± 10	52 ± 14	58 ± 5	1.00	0.15	0.18
LA dimension, mm	58 ± 9	55 ± 7	52 ± 9	0.59	0.06	0.41
LA area (2 chamber view), cm^2^	29 ± 9	25 ± 6	-	0.21	-	-
LA area (4 chamber view), cm^2^	28 ± 9	23 ± 6	-	0.07	-	-
LA volume (ml)	125 ± 54	92 ± 31	-	0.04		
RA dimension, mm	51 ± 10	52 ± 9	55 ± 9	0.98	0.42	0.58
LGE findings						
LV LGE	3 (15 %)	5 (27 %)	0 (0 %)	0.27	0.09	0.01
Papillary muscle LGE	0 (0 %)	0 (0 %)	0 (0 %)	-	-	-

*P value was calculated by one-way ANOVA with Tukey’s correction or Chi-square test

*ANOVA* analysis of variance, *CMR* cardiovascular magnetic resonance, *DBP* diastolic blood pressure, *DCM* dilated cardiomyopathy, *EDV* end-diastolic volume, *EDVI* end-diastolic volume index, *EF* ejection fraction, *ESV* end-systolic volume, *ESVI* end-systolic volume index, *HR* heart rate, *LGE* late gadolinium enhancement, *LV* left ventricle, *LVMI* left ventricular mass index, *MR* mitral regurgitation

**Table 3:** Comparison of papillary muscle related parameters

	DCM MR (+), *N* = 22	DCM MR (-), *N* = 18	Controls, *N* = 20	* P-value DCM MR (+) vs DCM MR (-)	* P-value DCM MR (+) vs Controls	* P-value DCM MR (-) vs Controls
Maximum anterior PAP diameter, mm	11.6 ± 3.3	10.2 ± 1.5	7.9 ± 1.3	0.14	<0.05	<0.05
Minimum anterior PAP diameter, mm	7.9 ± 1.9	7.6 ± 1.4	6.8 ± 0.5	0.73	<0.05	0.19
Maximum posterior PAP diameter, mm	10.6 ± 3.1	9.3 ± 1.5	7.0 ± 0.6	0.14	<0.05	<0.05
Minimum posterior PAP diameter, mm	6.9 ± 1.7	7.1 ± 0.9	5.0 ± 0.5	0.79	<0.05	<0.05
Maximum anterior PAP length, mm	44.7 ± 7.6	42.3 ± 6.3	34.5 ± 2.8	0.42	<0.05	<0.05
Minimum anterior PAP length, mm	37.6 ± 6.8	31.6 ± 5.4	23.5 ± 2.9	<0.05	<0.05	<0.05
Maximum posterior PAP length, mm	40.9 ± 8.4	37.8 ± 5.8	32.9 ± 3.1	0.28	<0.05	<0.05
Minimum posterior PAP length, mm	33.7 ± 7.3	27.9 ± 3.5	22.6 ± 3.1	<0.05	<0.05	<0.05
Anterior PAP shortening, %	15.8 ± 4.4	25.5 ± 4.1	32.0 ± 4.8	<0.05	<0.05	<0.05
Posterior PAP shortening, %	17.7 ± 5.8	26.0 ± 3.4	31.5 ± 4.9	<0.05	<0.05	<0.05
Mitral annulus (4chamber), mm	37.8 ± 6.6	32.5 ± 3.8	28.7 ± 1.1	<0.05	<0.05	<0.05
Mitral annulus (2chamber), mm	38.5 ± 4.5	34.2 ± 5.1	31.6 ± 2.0	<0.05	<0.05	<0.05
Tenting height, mm	10.3 ± 1.1	6.1 ± 1.7	2.7 ± 1.3	<0.05	<0.05	<0.05
Anterior mitral leaflet length, mm	23.6 ± 3.5	19.9 ± 2.3	21.7 ± 1.6	<0.05	0.06	0.11
Posterior mitral leaflet length, mm	13.9 ± 4.0	12.6 ± 1.9	10.4 ± 1.4	0.27	<0.05	<0.05

*P value was calculated by one-way ANOVA with Tukey’s correction

*ANOVA* analysis of variance, *DCM* dilated cardiomyopathy, *MR* mitral regurgitation, *PAP* papillary muscle

**Table 4:** Intra- and inter-observer variability for measurement of papillary muscle size

	Intra observer variability		Inter observer variability	
	ICC	95 % CI	ICC	95 % CI
Maximum papillary muscle diameter	0.93	0.84–0.97	0.92	0.81–0.97
Minimum papillary muscle diameter	0.87	0.69–0.94	0.82	0.61–0.93
Maximum papillary muscle length	0.91	0.78–0.96	0.88	0.73–0.95
Minimum papillary muscle length	0.91	0.78–0.96	0.86	0.70–0.95

*CI* confidence interval, *ICC* intra class correlation coefficients

### Comparison of papillary muscle T_1_ time between cardiomyopathy and healthy control subjects

**Fig. 3:**
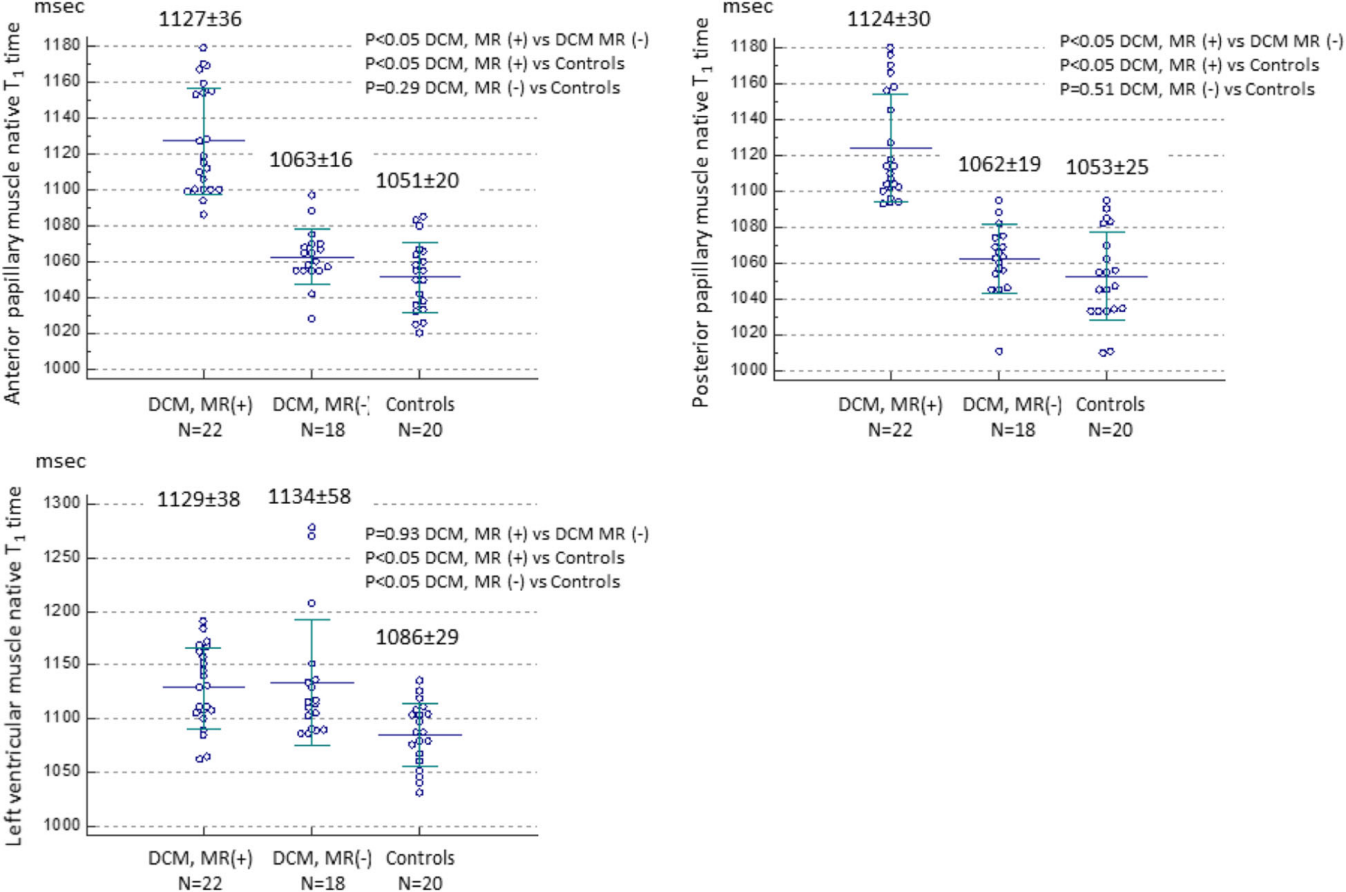
Comparison of papillary muscle native T_1_ time and left ventricular T_1_ time. Papillary muscle T_1_ time was significantly elevated in DCM patients with mitral regurgitation in comparison to DCM patients without mitral regurgitation and healthy controls. *DCM* dilated cardiomyopathy, *MR* mitral regurgitation

**Fig. 4:**
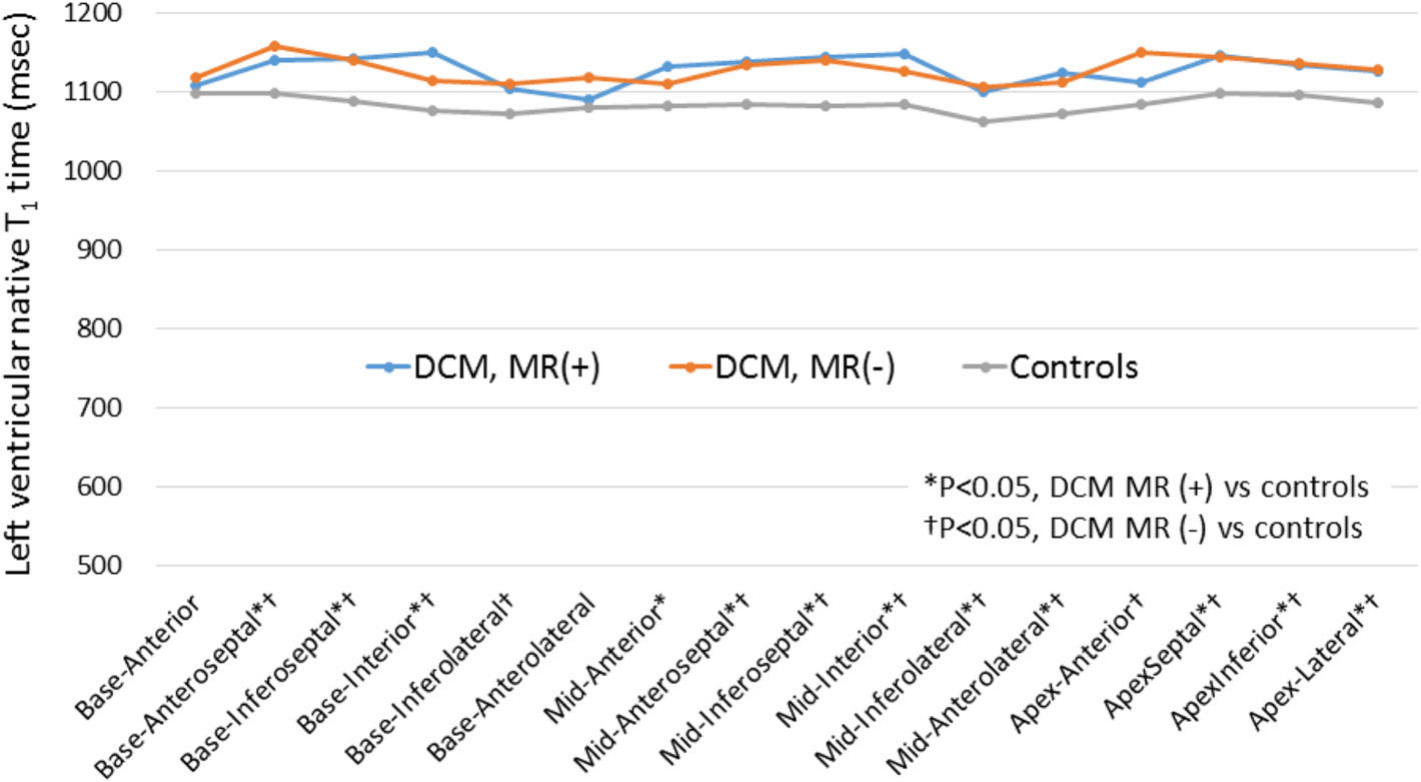
Regional left ventricular native T_1_ time in each segments. There was no substantial variability across 16 segments both in DCM patients and controls. *DCM* dilated cardiomyopathy, *MR* mitral regurgitation

**Fig. 5:**
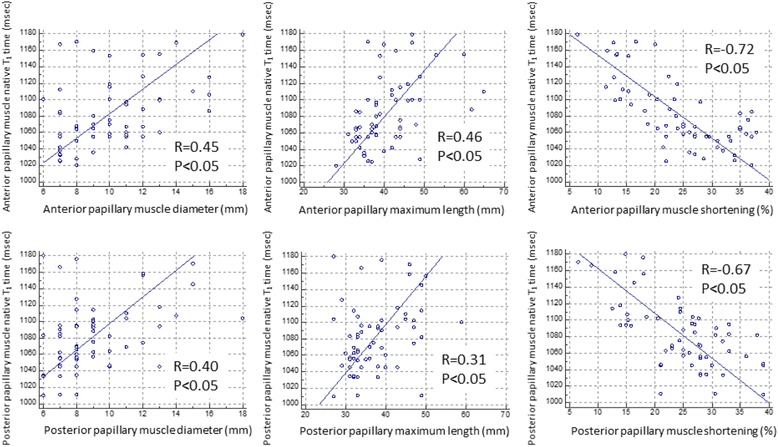
Relationship between papillary muscle native T_1_ time and morphology. Papillary muscle native T_1_ time was correlated with papillary muscle diameter, length and shortening

Figure 3 illustrates individual papillary muscle and LV T_1_ time for 3 groups. Mean anterior papillary muscle T_1_ time was 1127 ± 36 msec for DCM with mitral regurgitation (*p* < 0.05 vs DCM without mitral regurgitation; *p* < 0.05 vs healthy controls), 1063 ± 16 msec for DCM without mitral regurgitation (*p* = 0.29 vs healthy controls) and 1051 ± 20 msec for healthy controls. Mean posterior papillary muscle T_1_ time was 1124 ± 30 msec for DCM with mitral regurgitation (*p* < 0.05 vs DCM without mitral regurgitation; *p* < 0.05 vs healthy controls), 1062 ± 19 msec for DCM without mitral regurgitation (*p* = 0.51 vs healthy controls) and 1053 ± 25 msec for healthy controls. LV native T_1_ time was significantly elevated in DCM patients in comparison to healthy controls (*p* < 0.05, Fig. 3), but similar between DCM patients with mitral regurgitation and those without mitral regurgitation (1129 ± 38 msec vs 1134 ± 58 msec, *p* = 0.93). Figure 4 demonstrates regional LV native T_1_ time in each segments. There was no substantial variability across 16 segments both in DCM patients and controls. In addition, there was no significant correlation between papillary muscle T_1_ time and mid-level LV T_1_ time (*r* = 0.31, *p* = 0.05 by Spearman’s correlation coefficient). There was no significant difference in T_1_ time between base, mid and apical slices (base, 1125 ± 52 msec; mid, 1130 ± 47 msec; apex, 1138 ± 54 msec, *p* = 0.53 by one-way ANOVA). Figure 5 shows the relationship between papillary muscle T_1_ time and papillary muscle morphology. Elevated papillary muscle T_1_ time was associated with increased papillary muscle diameter, increased papillary muscle length and decreased papillary muscle shortening.

### Relationship between papillary muscle T_1_ time and mitral regurgitant fraction

**Fig. 6:**
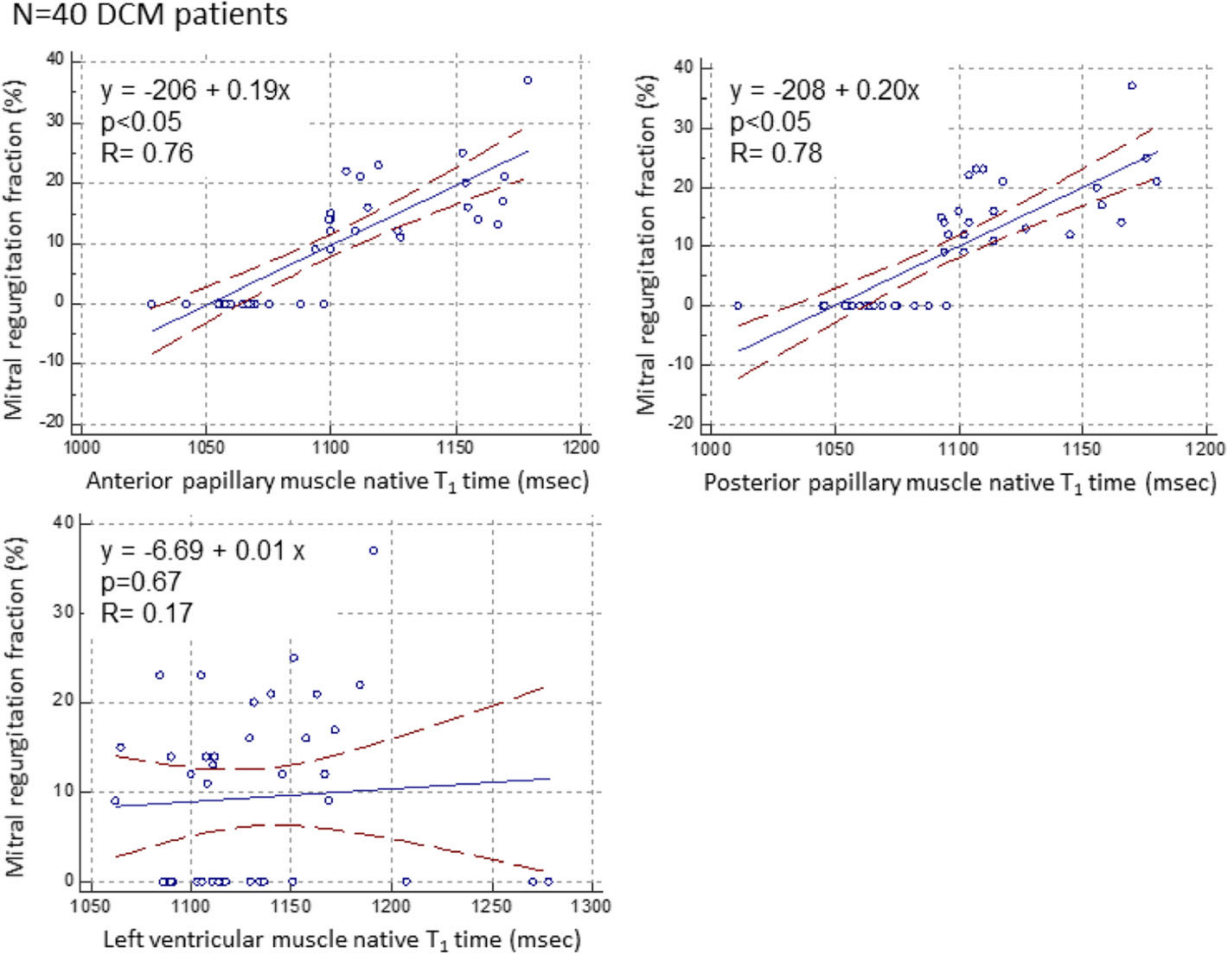
Relationship between papillary muscle native T_1_ time and mitral regurgitant fraction. Papillary muscle native T_1_ time was associated with mitral regurgitant fraction in DCM patients. *DCM* dilated cardiomyopathy

Figure 6 shows the relationship between papillary muscle T_1_ time and mitral regurgitant fraction in DCM patients. Mitral regurgitant fraction was significantly correlated with both anterior and posterior papillary muscle T_1_ time (*p* < 0.05) but not with LV myocardial T_1_ time (*p* = 0.67). Table 5 summarizes the results of multivariate linear regression analysis for determinants of mitral regurgitant fraction in all DCM patients (*n* = 40). In the multivariate analysis, average of anterior and posterior papillary muscle native T_1_ time was employed for analysis. Multivariate linear regression analysis identified papillary muscle native T_1_ time (β = 0.10, 95 % CI: 0.03–0.17, *p* < 0.05) as an independent determinant of mitral regurgitant fraction. Table 6 shows the results of multivariate linear regression analysis for determinants of mitral regurgitant fraction in DCM patients with MR (*n* = 22). Multivariable linear regression analysis identified posterior papillary muscle maximum diameter (β = 1.32, 95 % CI: 0.50–2.16, *p* < 0.05) and papillary muscle native T_1_ time (β = 0.11, 95 % CI: 0.03–0.20, *p* < 0.05) as independent determinants of mitral regurgitant fraction.

**Table 5:** Univariate and multivariate linear regression analysis for determinants of mitral regurgitant fraction in all DCM patients (*n* = 40)

	Univariable analysis			Multivariable analysis		
	B	95 % CI for β	*P*-value	B	95 % CI for β	*P*-value
Age, year	−0.01	−0.20–0.18	0.90	-	-	-
Gender, male	−3.99	−10.50–2.53	0.22	-	-	-
Body mass index, kg/m^2^	0.22	−0.21–0.71	0.28	-	-	-
LVEDVI, mL/m^2^	0.05	−0.02–0.12	0.18	-	-	-
LVEF, %	−0.16	−0.40–0.07	0.17	-	-	-
LVMI, g/m^2^	0.06	−0.08–0.20	0.37	-	-	-
Left atrial area (4 chamber), cm^2^	0.56	0.23–0.91	<0.05	0.17	−1.05–1.39	0.25
Left atrial area (2 chamber), cm^2^	0.36	−0.16–0.89	0.17	-	-	-
Left atrial volume, cm^3^	0.10	0.04–0.16	<0.05	−0.01	−0.21–0.20	0.95
Mitral annulus (4 chamber), mm	0.43	−0.01–0.87	0.06	-	-	-
Tenting height, mm	2.28	1.38–3.17	<0.05	0.61	−0.37–1.59	0.21
Anterior papillary muscle maximum diameter, mm	1.28	0.34–2.23	<0.05	0.29	−0.59–1.16	0.51
Posterior papillary muscle maximum diameter, mm	1.37	0.35–2.38	<0.05	0.73	−0.23–1.68	0.13
Anterior papillary muscle shortening, %	−0.87	−1.20– −0.54	<0.05	−0.10	−0.68–0.49	0.74
Posterior papillary muscle shortening, %	−0.92	−1.24– −0.60	<0.05	−0.21	−0.75–0.32	0.42
Papillary muscle T_1_ time, sec	0.16	0.12–0.21	<0.05	0.10	0.03–0.17	<0.05

Variables with *p* value < 0.05 in univariate analysis were included in multivariable analysis

*CI* confidence interval, *DCM* dilated cardiomyopathy, *LVEDVI* left ventricular end-diastolic volume index, *LVEF* left ventricular ejection fraction, *LVMI* left ventricular mass index, *MR* mitral regurgitation

**Table 6:** Univariate and multivariate linear regression analysis for determinants of mitral regurgitant fraction in DCM patients with MR (*n* = 22)

	Univariable analysis			Multivariable analysis		
	B	95 % CI for β	*P*-value	B	95 % CI for β	*P*-value
Age, year	0.08	−0.11–0.26	0.41	-	-	-
Gender, male	−0.49	−6.85–5.86	0.87	-	-	-
Body mass index, kg/m^2^	0.17	−0.29–0.64	0.44	-	-	-
LVEDVI, mL/m^2^	0.05	−0.02–0.11	0.17	-	-	-
LVEF, %	−0.17	−0.40–0.05	0.12	-	-	-
LVMI, g/m^2^	0.06	−0.06–0.20	0.28	-	-	-
Left atrial area (4 chamber), cm^2^	0.34	0.01–0.68	<0.05	0.13	−0.24–0.30	0.85
Left atrial area (2 chamber), cm^2^	0.14	−0.21–0.49	0.42	-	-	-
Left atrial volume, cm^3^	0.05	−0.01–0.11	0.08			
Mitral annulus (4 chamber), mm	−0.05	−0.50–0.41	0.83	-	-	-
Tenting height, mm	1.46	−1.09–4.01	0.24	-	-	-
Anterior papillary muscle maximum diameter, mm	0.64	−0.22–1.51	0.14	-	-	-
Posterior papillary muscle maximum diameter, mm	0.93	0.05–1.82	<0.05	1.32	0.50–2.16	<0.05
Anterior papillary muscle shortening, %	0.31	−1.12– −0.21	0.17	-	-	-
Posterior papillary muscle shortening, %	−0.51	−0.97– −0.05	<0.05	−0.17	−0.58–0.23	0.41
Papillary muscle T_1_ time, sec	0.11	0.02–0.20	<0.05	0.11	0.03–0.20	<0.05

Variables with *p* value < 0.05 in univariate analysis were included in multivariable analysis

*CI* confidence interval, *DCM* dilated cardiomyopathy, *LVEDVI* left ventricular end-diastolic volume index, *LVEF* left ventricular ejection fraction, *LVMI* left ventricular mass index, *MR* mitral regurgitation

### Variability of papillary muscle T_1_ measurement

Repeatability coefficients of anterior papillary muscle T_1_ time were 2.0 % for intra-observer variability, 4.1 % for inter-observer variability. Repeatability coefficients of posterior papillary muscle native T_1_ time were 1.3 % for intra-observer variability, 5.0 % for inter-observer variability. Variability of papillary muscle native T_1_ time measurement was high (CV of intra-observer variability: 0.9 % for anterior papillary muscle and 0.7 % for posterior papillary muscle; CV of inter-observer variability: 1.6 % for anterior papillary muscle and 1.8 % for posterior papillary muscle).

## Discussion

To the best of our knowledge, this study is the first investigation to examine the feasibility and variability of papillary muscle native T_1_ time measurement, and to investigate the relationship between papillary muscle T_1_ time and severity of functional mitral regurgitation in DCM patients. We found a significant difference of papillary muscle native T_1_ time between DCM patients with mitral regurgitation and those without mitral regurgitation, a correlation between papillary muscle native T_1_ time and mitral regurgitant fraction in DCM patients and low variability of papillary muscle T_1_ time measurement.

### Papillary muscle T_1_ time and papillary muscle morphology in DCM patients

Previous echocardiographic studies have shown that the papillary muscle dysfunction is observed in several cardiovascular diseases. Papillary muscle shortening has been assessed using transthoracic echocardiography and used as a functional parameter [24, 25]. In myocardial infarction patients, papillary muscle function was substantially reduced compared to healthy controls (papillary muscle shortening: 15 ± 14 % vs 30 ± 8 %) [25]. Reduced papillary muscle shortening has also been reported in cardiomyopathy patients including hypertrophic cardiomyopathy or DCM [24, 26]. In the current study, we measured papillary muscle diameter, length and shortening using cine CMR and showed that papillary muscle shortening was significantly reduced in DCM patients in comparison to healthy controls. This absolute value and difference findings were consistent with previous echocardiographic studies. In addition, we found a significant difference in papillary muscle shortening between DCM patients with mitral regurgitation in comparison to those without mitral regurgitation, suggesting that papillary muscle dysfunction may be contributing to mitral regurgitation.

The maximum papillary muscle diameter was approximately 7 mm in healthy controls and 10 mm in DCM patients. Elevated papillary muscle native T_1_ time was associated with larger diameter, longer length, decreased shortening in DCM patients. This finding suggested that papillary muscle T_1_ time might reflect papillary muscle pathological changes in DCM patients (i.e. papillary muscle fibrosis). Furthermore, papillary muscle native T_1_ time was increased in DCM patients with mitral regurgitation compared to those without mitral regurgitation, but LV native T_1_ time was similar between 2 groups. These results suggest that the papillary muscle native T_1_ time may be a more sensitive for the mechanical stress induced by functional mitral regurgitation. A previous study by Okayama et al. showed that LGE evidence of bilateral papillary muscle infarction correlated with LV remodeling and functional mitral regurgitation [27]. Although the assessment of papillary muscle abnormality is feasible by LGE, an important advantage of non-contrast native T_1_ mapping is the ability to evaluate papillary muscle abnormality in patients with renal dysfunction who are at high risk of systemic nephrogenic fibrosis [28]. In addition, we did not observe any papillary muscle LGE in this study, suggesting that the papillary muscle pathological change is *diffuse* rather than *focal* in DCM patients.

### Clinical implication

We found significant difference in papillary muscle native T_1_ time between DCM patients with mitral regurgitation and those without mitral regurgitation. However, no significant difference was found in LV native T_1_ time. In addition, papillary muscle T_1_ time was independently correlated with severity of functional mitral regurgitation after adjustment of conventional determinants, including tenting height, mitral annulus diameter. Previous transthoracic echocardiographic studies showed that tenting height is a strong indicator of effective orifice area in patents with LV dysfunction and functional mitral regurgitation [29]. Further study is necessary to elucidate if papillary muscle native T_1_ time has prognostic value in DCM patients.

### Study limitations

Our study has several limitations. The sample size was modest and the study population was limited to DCM patients and healthy controls. Our T_1_ mapping sequence, STONE, has not been histologically validated for papillary muscle T_1_ mapping. Therefore, we do not know the true cause of the elevated papillary muscle native T_1_ time in DCM patients. Although T_1_ mapping images acquired by STONE were motion corrected, relatively low spatial resolution of T_1_ mapping images and the partial volume effect are not negligible for T_1_ measurement of papillary muscle. To avoid partial volume effect with blood pool, we’ve carefully placed the ROI on papillary muscle much smaller than actual papillary muscle diameter not to include the pixels from LV blood pool. As shown on Fig. 2, size of ROI on papillary muscle was much smaller than actual size of papillary muscle. The pixel size of ROI drawn on papillary muscle was 16–40 pixels. It would be also interesting to investigate the relationship between papillary muscle extra cellular volume (ECV) and mitral regurgitant fraction. However, calculation of ECV requires registration between native and post-contrast T1 images, which will be challenging for a mobile and small papillary muscle anatomy. Regarding LV ECV, in our cohort, there were 21 DCM patients and 9 controls with ECV data. Significant difference was found between DCM patients and controls in LV ECV averaged over 16 segments (0.30 ± 0.04 vs 0.27 ± 0.02, *p* = 0.03). However, no significant difference was found between DCM patients with MR and those without MR (0.31 ± 0.05 vs 0.29 ± 0.04, *p* = 0.63). A larger, more diverse study is required to assess the clinical relevance of papillary muscle native T_1_ time. Because this study is a cross-sectional study, we can’t say any causal relationship between papillary muscle T_1_ time and functional mitral regurgitation. Examination of the potential difference of the time course of T_1_ change in the myocardium and papillary muscle would also be of interest, but is beyond the scope of this study.

## Conclusions

Measurement of papillary muscle native T_1_ time is both feasible and reproducible. This CMR approach successfully detects abnormal papillary muscle native T_1_ time in DCM patients with functional mitral regurgitation.

## Abbreviations

ANOVA: Analysis of variance
ARCTIC: Adaptive registration of varying contrast-weighted images for improved tissue characterization
CI: Confidence interval
CMR: Cardiovascular magnetic resonance
CV: Coefficient of variation
DCM: Dilated cardiomyopathy
ECV: Extracellular volume
FA: Flip angle
FOV: Field of view
LA: Left atrial
LGE: Late gadolinium enhancement
LV: Left ventricular
MR: Mitral regurgitation
ROI: Region of interest
SD: Standard deviation
SENSE: Sensitivity encoding
SSFP: Steady-state free precession
STONE: Slice-interleaved T1
TE: Echo time
TFE: Turbo field echo
TR: Repetition time
